# Re-188 Enhances the Inhibitory Effect of Bevacizumab in Non-Small-Cell Lung Cancer

**DOI:** 10.3390/molecules21101308

**Published:** 2016-09-30

**Authors:** Jie Xiao, Xiaobo Xu, Xiao Li, Yanli Li, Guobing Liu, Hui Tan, Hua Shen, Hongcheng Shi, Dengfeng Cheng

**Affiliations:** 1 Department of Nuclear Medicine, Zhongshan Hospital, Fudan University, Shanghai 200032, China; xiaojie_fdu@163.com (J.X.); leelobster@126.com (X.L.); drliyanli@163.com (Y.L.); liuguobing0422@163.com (G.L.); 13818914579@163.com (H.T.); shihongcheng163@163.com (H.S.); 2 Shanghai Institute of Medical Imaging, Shanghai 200032, China; 3 Departments of Respiratory Medicine, Zhongshan Hospital, Fudan University, Shanghai 200032, China; xu.xiaobo@zs-hospital.sh.cn; 4 Institute of Applied Physics, Chinese Academy of Sciences, Shanghai 200032, China; shenhua@sinap.ac.cn

**Keywords:** bevacizumab, non-small cell lung cancer, radioimmunotherapy, Re-188, Tc-99m

## Abstract

The malignant behaviors of solid tumors such as growth, infiltration and metastasis are mainly nourished by tumor neovascularization. Thus, anti-angiogenic therapy is key to controlling tumor progression. Bevacizumab, a humanized anti-vascular endothelial growth factor (VEGF) antibody, plus chemotherapy or biological therapy can prolong survival for cancer patients, but treatment-related mortality is a concern. To improve inhibitory effect and decrease side-effects on non-small-cell lung cancer (NSCLC), we used Re-188, which is a β emitting radionuclide, directly labeled with bevacizumab for radioimmunotherapy in a human A549 tumor model. Cytotoxic assay data showed that, after ^188^ReO_4_^−^ or ^188^Re-bevacizumab at different concentration for 4 and 24 h, a time- and radioactivity does-dependent reduction in cell viability occurred. Also, an apoptosis assay conformed great apoptosis in the ^188^Re-bevacizumab group compared with controls and other treatment groups. In vivo, tumor volumes in the ^188^Re-bevacizumab (11.1 MBq/mice) group were not reduced but growth was delayed compared with other groups. Thus, ^188^Re-bevacizumab enhanced the therapeutic effect of bevacizumab, suggesting a potential therapeutic strategy for NSCLC treatment.

## 1. Introduction

Lung cancer is the leading cause of cancer death worldwide and represents a significant financial and social burden [1]. Non-small-cell lung cancer (NSCLC), the mostly common cancer subtype, accounts for 85% of all new lung cancer diagnoses [2]. Due to late onset of clinical manifestations, patients are commonly diagnosed at advanced or inoperable-stages. Recently, chemotherapy with platinum plus one other anti-tumor agents is recommended for first-line chemotherapy, but side-effects are a concern [3]. Although maintenance therapy followed by second-line cytotoxic chemotherapy is common, advanced stage patients did not live one year beyond median survival [4]. Thus, effective, low side-effect therapeutic methods for NSCLC treatment are needed.

Tumor progression (growth, infiltration and metastasis) are reportedly supported by tumor neovascularization. Thus, tumor angiogenesis is thought to be an approach for treating cancers [5,6]. Bevacizumab, a humanized monoclonal antibody (MAb), that specifically binds to vascular endothelial growth factor (VEGF) and can block neovascularization, was approved by the FDA [7]. In randomized clinical trials, survival benefits were improved when bevacizumab was added to standard chemotherapy regimens to treat metastatic colorectal cancer and advanced NSCLC [7,8]. Radioimmunotherapy (RIT) is an alternative for patients with advanced cancers. Using selective targeting to cancer-associated antigens on the tumor-cell surface, monoclonal antibody-mediated radionuclide can deliver high-dose therapeutic radiation to cancer cells with minimal exposure of normal cells [9,10,11]. The first radiolabeled antibody for anti-tumor therapy, ^90^Y-murine anti-CD20 antibody, ibritumomab, offered a satisfactory therapeutic response, and was approved for clinical practice in 2002 [12]. Coupled to ^131^I, tositumomab has been used to treat chronic lymphocytic leukemia or small lymphocytic lymphoma in first remission. Also, patients with leukemias or lymphomas enjoyed more survival benefits from RIT than without RIT treatment [13,14,15]. However, for solid tumor, which are radio-resistant and less accessible to MAbs, a concern remains about limited clinical efficacy [16].

According to Lucas, ^90^Y and ^188^Re were regarded as the best candidates for solid tumor treatment such as for NSCLC [17]. In addition, considering the appropriate half-life of 16.9 h (0.7 d), therapeutic beta radiation (Eβ = 2.118 Mev) and easy used in-home, ^188^Re was selected as the radioisotope for RIT in this study. The radiotherapeutic agent ^188^Re-bevacizumab was designed, and its therapeutic efficacy was evaluated in tumor models of NSCLC.

## 2. Results and Discussion

### 2.1. Radiolabeling

**Figure 1:**
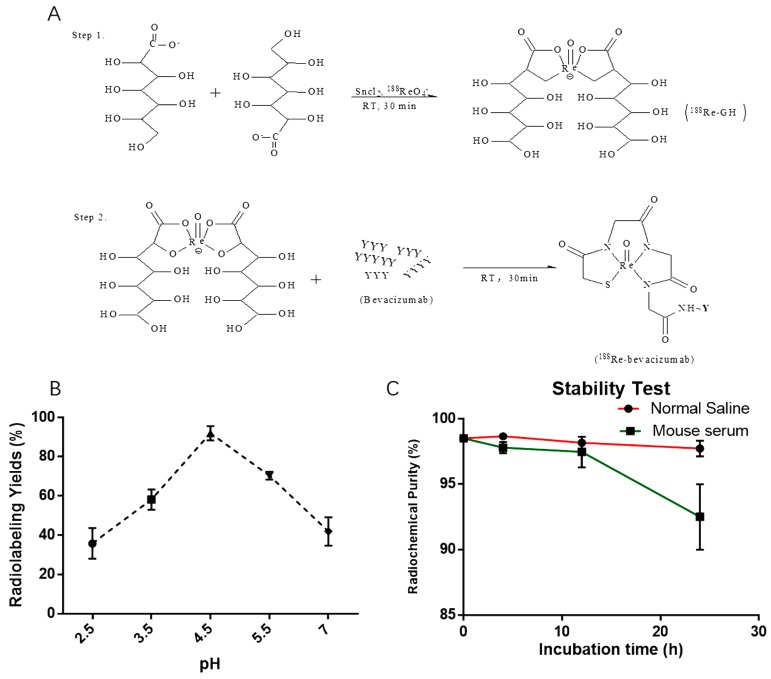
(**A**) Synthesis of ^188^Re-bevacizumab by a two-step reaction; (**B**) Effects of pH on complexation yield of ^188^Re-bevacizumab; (**C**) Stability of ^188^Re-bevacizumab incubated with normal saline (pH 7.4) and mouse serum in vitro at room temperature was measured at various times.

The synthesis of ^188^Re-bevacizumab was achieved by a two-step reaction (Figure 1A). For paper chromatography studies, the Rf values of radiolabeled bevacizumab and ^188^Re-colloid were 0.0–0.1, and the Rf value of free or unbound ^188^ReO_4_^−^ was 0.8–0.9 in normal saline. In contrast, for the mixed solvent system v_ethanol_:v_ammonia_:v_water_ = 2:5:1 the Rf value of radiolabeled bevacizumab and free ^188^ReO_4_^−^ were 0.8–0.9, and the Rf value of radiolabeled bevacizumab and ^188^Re-colloid were 0.0–0.1. Thus, yields of 94.5% ± 1% were calculated for unbound ^188^ReO_4_^−^ and less than 2% radiocolloidal under these conditions: 100 μg (1 mg/mL) of bevacizumab was incubated with ^188^ReO_4_^−^ (37–74 MBq) in acetic acid buffer solution (pH = 4.5) for 30 min at room temperature (RT). Purification was performed with disposable G25 PD-10 desalting columns (GE Healthcare Bio-Sciences Corp., Piscataway, NJ, USA) and a radiochemical purity of more than 98% was achieved. After purification, radiolabeled products were used for studies.

In this present study, the influence on radiolabeling efficiency by pH was also investigated. The optimal pH value was 4.5, as the results in Figure 1A. According to the published literature [18,19,20], the direct radiolabeling of ^188^Re to antibodies occurs within a pH range from 4 and 6, and it is proposed that a radiolabeling pH value lower than 4 could compromise the immunoreactivity of the antibodies, while a higher pH value increases the percentage of unbound ^188^ReO_4_^−^.

### 2.2. Stability in Vitro

After purification, ^188^Re-bevacizumab was diluted and incubated in mouse serum and normal saline (1:5 and 1:1, respectively). Radiolabeling was measured at 4, 12 and 24 h at 37 °C (Figure 1C). After 12 h of incubation, over than 95% of labelled products remained intact in both normal saline and serum. For 24 h, less than 10% decomposition was observed in mouse serum, suggesting a favorable stability in vitro.

### 2.3. Cytotoxicity Assay (CCK-8)

**Figure 2:**
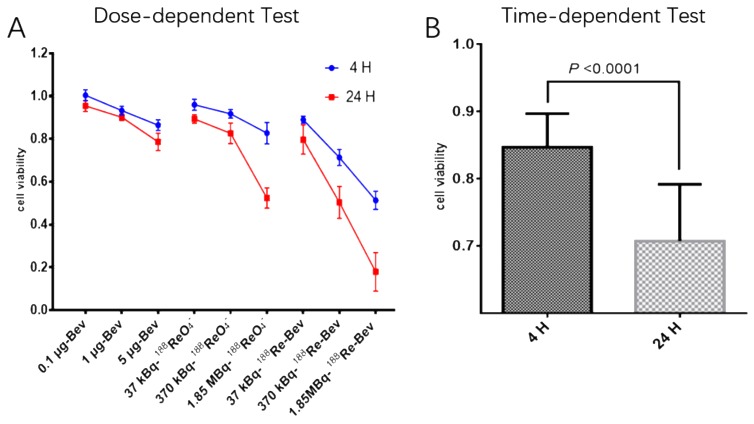
Cell viability is in a radioactive- and time-dependent manner. (**A**) Dose-dependent test: changes of cell viability after incubation with fresh medium containing bevacizumab at various concentrations, free ^188^Re and ^188^Re-bevacizumab; (**B**) Time-dependent test: compared average cell viability of all treated group (bevacizumab, free ^188^Re and ^188^Re-bevacizumab) at 4 h and at 24 h.

Cell viability of all treated groups at different times points is shown in Figure 2, where the data show that the viability was time- and dose- or radioactive dose-dependent. Cells incubated with fresh medium were the most viable, whereas ^188^Re-bevacizumab (1.85 MBq) treatment reduced viability the most.

Increasing the radioactivity dose from 37 kBq to 1.85 MBq proportionally decreased cell viability at 4 h and 24 h in all groups. Thus, A549 cells are more sensitive to ^188^Re-bevacizumab compared to bevacizumab alone at equal concentration. How bevacizumab inhibits cell growth is unknown, but Wang suggested that cytotoxicity in tumor cells after bevacizumab administration may be due to endoplasmic reticulum stress, which accelerates apoptosis, or autocrine VEGF [21].

### 2.4. Apoptosis Assay 

**Figure 3:**
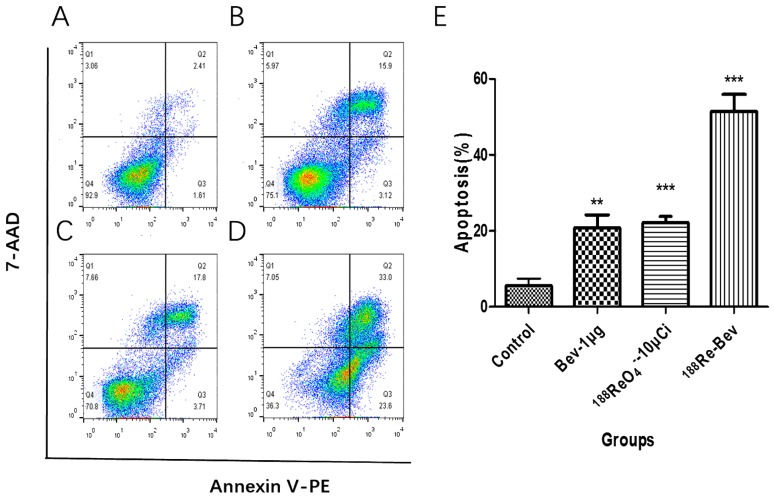
The percentage of apoptotic cells, inducing by bevacizumab (**B**); unbound ^188^ReO_4_^−^ (**C**) and ^188^Re-bevacizumab (**D**) at equal concentration or radioactivity, untreated group as control (**A**). (**E**) The combined pro-apoptotic efficiency of ^188^Re-bevacizumab is greater than the sum if each agent used along. ** *p* < 0.01; *** *p* < 0.001.

Viable cells were identified as PE-Annexin V^−^ and 7-AAD^−^ (lower left quadrants, Q4) and PE-Annexin V^−^ and 7-AAD^+^ (upper left quadrants, Q1) were necrotic. Early apoptotic cells were PE-Annexin-V^+^ and 7-AAD^−^ (lower right quadrants, Q3), while PE-Annexin-V^+^ and 7-AAD^+^ (upper right quadrants, Q2) were late apoptotic cells. The percentages of early apoptotic cells and late apoptotic cells from each group appear in Figure 3. Compared with controls (4.02% ± 1.05%), apoptosis increased after bevacizumab treatment, ^188^ReO_4_^−^ (370 kBq /mL), and ^188^Re-bevazucimab (370 kBq/μg/mL).

### 2.5. Micro SPECT/CT Imaging

**Figure 4:**
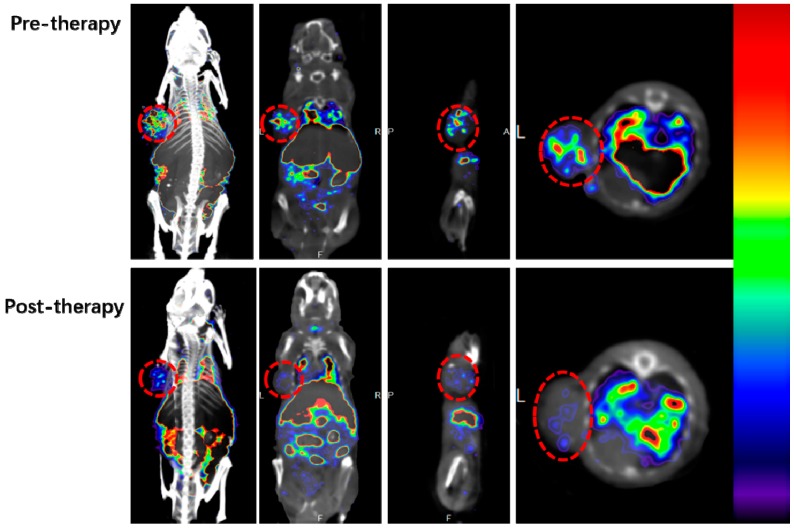
Maximum Intensity Projection (MIP) images and its axial, transverse and sagittal images demonstrate that ^99m^Tc-bevacizumab uptake in tumor are less than pre-therapy.

To eliminate the influence on therapeutic efficiency by ^188^ReO_4_^−^ before and after treatment, ^99m^Tc-MAG_3_-bevacizumab was used to monitor angiogenic changes during treatment. Whole body images and relevant axial slices of pre- and post-therapy A549 xenograft mouse treated with ^188^Re-bevacizumab are depicted in Figure 4. Images acquired at 4 h after injection showed intense uptake of drug by lung, liver, spleen, kidney and bladder. Tumor uptake before treatment was more intensive than post-treatment, suggesting effective anti-angiogenic efficacy of ^188^Re-bevacizumab, and treatment was ceased. Bevacizumab has a long half-life and binds to VEGF-A, a key mediator of angiogenesis [22]. Thus, the accumulation of ^99m^Tc-MAG_3_-bevacizumab in organs may be explained by organ mesenchyme secretion of VEGF [23].

### 2.6. Assessment of Treatment Effect in Vivo

**Figure 5:**
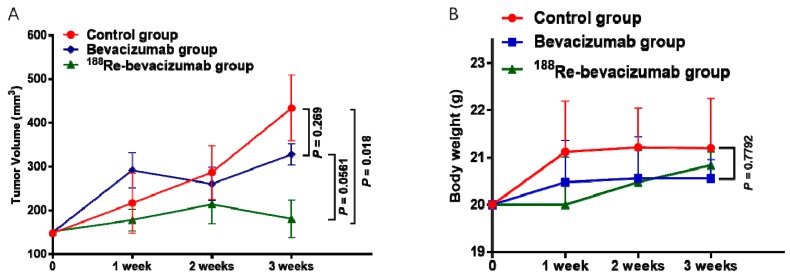
The alteration of tumor volumes and body weight. (**A**) Tumor volume are effectively inhibited in ^188^Re-bevacizumab group; (**B**) No significant weight loss is occurred in ^88^Re-bevacizumab group.

Bevacizumab (1.5 mg/kg or 30 μg per mice) and ^188^Re-bevacizumab (11.1 MBq/30 μg per mice) were separately injected into the tail vein treatment groups weekly and tumor sizes and body weight were recorded (Figure 5).

Compared with controls, tumor growth in the ^188^Re-bevacizumab and bevacizumab treatment groups were obviously delayed with no significant regression (*p* value = 0.018 < 0.05). Although there was no statistical difference (*p* value = 0.0561 > 0.05) between the ^188^Re-bevacizumab treatment group and the bevacizumab treatment group, more pronounced inhibition was observed in the ^188^Re-bevacizumab treatment group. According to a previous report [24], dosimetry analyses of ^177^Lu-DOTA-RS7 showed that complete remissions were obtained in the maximal tolerated dose group in tumor-bearing nude mice. Thus, in order to gain more benefit from ^188^Re-bevacizumab treatment, dosimetry analyses will be performed in our future work. Animal weight gain one week after treatment in the treatment groups was slowed and food intake was less, but these differences were not statistically different (*p* value = 0.7792 > 0.05).

Effective radiotherapy requires maximum therapeutic radioactivity accumulation in tumors and long-time retention. Micro SPECT/CT images show that ^99m^Tc-bevacizumab was taken up by tumors and cleared slowly. Similarly characteristics of bevacizumab permit the delivery of a high-dose ^188^Re to tumor tissues and minimizes exposure of normal tissue.

## 3. Experimental Section

### 3.1. Materials and Reagents

Solvents and regents used in radiolabeling reactions were of analytical grade, used without any further purification and purchased from Sigma-Aldrich (St. Louis, MO, USA), BD Biosciences (New York, NJ, USA). Bevacizumab (Avastin) was from Roche Pharma Ltd. (Basel, Switzerland).

^188^Re was obtained as its perrhenate by elution from an alumina-column-based ^188^W/^188^Re generator with normal saline (LaiTai Biotechnology, Jiangsu, China). Na^99m^TcO_4_ was supplied by Shanghai GMS Pharmaceutical Co., Ltd. (Shanghai, China). Flexible silica gel 60 F254 plates was purchased from Merck (Darmstadt, Germany) and used for thin-layer chromatography (TLC) studies. Micro SPECT/CT imaging was performed with a Nano SPECT/CT scanner (Mesido, Budapest, Hungary) at the Cancer Center of Shanghai.

Human lung A549 adenocarcinoma cells were purchased from the Chinese Type Culture Collection (Chinese Academy of Sciences, Shanghai, China). Male nude mice (4–6 weeks-old, 20–25 g) obtained from Slac Biotechnology (Shanghai, China) which were bred in a SPF laboratory animal facility at Fudan University were used as xenograft models. All animal experiments were conducted in accordance with relevant guidelines and regulations, and were approved by the Institutional Animal Care and Committee of Fudan University.

### 3.2. Radiolabeling and Stability in Vitro

#### 3.2.1. Radiolabeling

Bevacizumab, monoclonal antibody, was directly labeled with ^188^ReO_4_^−^ by ligand exchange using ^188^Re-glucoheptonate according to a published method [25,26]. Briefly, 100–200 μL of ^188^ReO_4_^−^ fresh perrhenate eluate (185–370 MBq) in normal saline were added to a solution containing 10 mg of sodium α-d-glucoheptonate dihydrate (dissolved in acetic acid, pH value of 2.5–7, 0.1 M), and treated with SnCl_2_ (range 200–1500 μg) as reductant at room temperature for 30 min. The ^99m^Tc-MAG_3_-bevacizumab radioproduct was synthesized according to described procedures [27,28].

#### 3.2.2. Stability Study in Vitro

To estimate the stability of ^188^Re-bevacizumab in vitro, a sample (10 μL) from the final preparation was incubated in normal saline (10 μL) and diluted in mice serum (50 μL) at 4 h,12 h and 24 h at 37 °C. The analysis was repeated three times.

Labeling efficiency and stability was measured by spotting samples on BSA blocked instant thin-layer chromatography silica gel (ITLC-SG). When the eluent of normal saline reached the front, the strip was removed, dried and cut into 1-cm sections and counted. All radioactive measurements were conducted on a gamma radioimmunoassay counter (GC-1200, Zhongjia Co., Ltd., Hefei, China). Radiolabeling of ^188^Re-bevacizumab = 100% − colloidal (%) − unbound ^188^ReO_4_^−^ (%).

### 3.3. Cytotoxicity Assay (CCK-8)

The human adenocarcinoma A549 cell line was cultured in Dulbecco‘s Modified Eagle Media (DMEM) or RPMI-1640 medium supplemented with 10% fetal bovine serum (Gibco, Life Tech, Grand Island, NE, USA), 2 mM l-glutamine, penicillin (100 IU/mL) and streptomycin (100 μg/mL) in a humidified atmosphere of air containing 5% CO_2_ at 37 °C. A549 cells cultured at the logarithmic growth phase were passaged or stored at 1:3 ratios. All experiments were performed between passages 3 and 5.

Cytotoxicity of ^188^Re-bevacizumab was evaluated with a cell count kit-8 (CCK-8) according to the manufacturer’s instruction. A549 cells were trypsinized and suspended at a concentration of 5 × 10^4^/mL and when they reached 80% confluence, 5 × 10^3^ cell were seeded and cultured with 100 μL complete medium with 10% FBS in 96-well plates coated with 0.05% rat tail collagen. After culturing for 24 h, medium was removed and replaced with 100 μL fresh medium containing bevacizumab at different concentrations, ^188^Re (1, 10, 50 μCi/mL) and equal amounts of ^188^Re-bevacizumab. A 549 cells were incubated in each desired medium as stated above for 4 h and 24 h. To evaluate cell viability, 10 μL of CCK-8 solution was added to each well, and 96-well plates were continuously incubated at 37 °C for 2 h. The OD values were read at 450 nm to measure cell viability as following: cell viability (%) = [OD (treated group) − OD (blank)] / [OD (control group) − OD (blank)] × 100%.

### 3.4. Apoptosis Assay

An apoptosis kit (FITC Annexin V Apoptosis Detection Kit) was used to measure apoptotic and necrotic cells. According to the manufacturer’s instructions, A549 cells were seeded in 25 cm^2^ plastic flasks and incubated for 48 h. Then, cells were treated with bevacizumab (1 μg), ^188^Re (370 kBq or 10 μCi), ^188^Re-bevacizumab (10 μCi-1 μg), meanwhile, cells incubated with fresh medium were controls. Next, 24 h later, cells were collected, washed twice with cold PBS, and then re-suspended and diluted in 1 × binding buffer (1 ×10^6^/mL). Then, 1 × 10^5^ cells (100 μL) were stained with PE-Annexin V (5 μL) and 7-AAD (5 μL) and incubated for 15 min at room temperature in the dark. Experiments were performed in triplicate.

### 3.5. Tumor Xenografts and Treatment Design

Tumor xenografts were established in nu/nu male mice via injection of approximately 6 × 10^6^ A549 cells in the left shoulder area. When tumor volumes reached 150 mm^3^, mice were randomly divided into three groups of fifteen animals as follows: controls, bevacizumab treated, and ^188^Re-bevacizumab treated. Furthermore, A549 tumor models were treated weekly for three weeks as follows: (1) normal saline every week (control group, 100 μL i.v.); (2) bevacizumab (1.5 mg/kg/week, 100 μL, i.v.); (3) ^188^Re-bevacizumab (11.1 MBq/week/mouse, 100 μL, i.v.); Tumor volumes and animal weights were recorded weekly. Tumor volumes were estimated using the equation of 0.5 × length × (width)^2^. All animals were observed daily to assess general health.

### 3.6. Micro SPECT/CT Imaging

Micro-SPECT/CT imaging was following the described method [29] in three mice before the initial treatment with ^188^Re-bevacizumab and after final treatment at 3 weeks. ^99m^Tc-MAG_3_-bevacizumab (500 μCi/50 μg/mice) was injected, and then CT and SPECT imaging was performed 4 h later.

## 4. Conclusions

^188^Re-bevacizumab offered high yield with direct labeling and had favorable stability in vitro. ^188^Re-bevacizumab enhanced tumor inhibition for NSCLC in both in vivo and in vitro studies.
